# Prediction of drug-target interactions based on substructure subsequences and cross-public attention mechanism

**DOI:** 10.1371/journal.pone.0324146

**Published:** 2025-05-30

**Authors:** Haikuo Shi, Jing Hu, Xiaolong Zhang, Shuting Jin, Xin Xu

**Affiliations:** Jing Hu, School of Computer Science and Technology, Wuhan University of Science and Technology, Wuhan, Hubei, China; University of Milano–Bicocca: Universita degli Studi di Milano-Bicocca, ITALY

## Abstract

Drug-target interactions (DTIs) play a critical role in drug discovery and repurposing. Deep learning-based methods for predicting drug-target interactions are more efficient than wet-lab experiments. The extraction of original and substructural features from drugs and proteins plays a key role in enhancing the accuracy of DTI predictions, while the integration of multi-feature information and effective representation of interaction data also impact the precision of DTI forecasts. Consequently, we propose a drug-target interaction prediction model, SSCPA-DTI, based on substructural subsequences and a cross co-attention mechanism. We use drug SMILES sequences and protein sequences as inputs for the model, employing a Multi-feature information mining module (MIMM) to extract original and substructural features of DTIs. Substructural information provides detailed insights into molecular local structures, while original features enhance the model’s understanding of the overall molecular architecture. Subsequently, a Cross-public attention module (CPA) is utilized to first integrate the extracted original and substructural features, then to extract interaction information between the protein and drug, addressing issues such as insufficient accuracy and weak interpretability arising from mere concatenation without interactive integration of feature information. We conducted experiments on three public datasets and demonstrated superior performance compared to baseline models.

## Introduction

Accurately predicting drug-target interactions (DTI) is essential for drug discovery and repurposing. While conventional experimental techniques in the laboratory are still extremely reliable, they are also notably time-consuming and require significant manual effort. Researchers must conduct extensive chemical and biomedical experiments in the lab, screening from a large pool of drugs, while also facing issues such as limited data acquisition and poor scalability. Meanwhile, researchers have begun to apply various machine learning methods to DTI prediction and have made significant progress. For example, Support Vector Machines (SVM) [1] and Random Forest (RF) [2,3]. However, traditional machine learning models have relatively limited performance when dealing with complex nonlinear relationships. The interactions between biomolecules are often highly complex nonlinear processes, which may prevent traditional machine learning models from capturing this complexity, thus limiting the accuracy and adaptability of DTI predictions.

Deep learning models generally exhibit better performance than traditional machine learning [4–6] models because they can learn complex nonlinear relationships. They are particularly suited for describing intricate interactions between biomolecules, making them highly adaptable to high-throughput data in the biomedical field.

Similar to using textCNN for semantic learning of word sequences, Huang et al. [7] proposed an model, MolTrans, which takes drug and protein sequences as inputs. By incorporating a transformer encoder, the model captures the interaction information between drugs and proteins in greater detail, making the interaction maps produced by the model more interpretable. However, when considering drug-target interactions, the model only uses substructural information and overlooks the original features of DTIs. The more global information contained in the original features is not fully utilized, thus limiting the model’s understanding of the molecular overall structure. Ozturk et al. [8] introduced DeepDTA, which utilizes two distinct convolutional neural network (CNN) blocks to separately process SMILES strings and protein sequences. This approach aids in capturing local features within protein and drug sequences, enhancing the model’s ability to model complex relationships. Bai et al. [9] developed DrugBAN, which leverages graph convolutional networks (GCNs) and one-dimensional convolutional neural networks (1D-CNNs) to extract substructural features from drug molecular graphs and protein sequences, respectively. Subsequently, a bilinear attention network module explicitly learns the local interaction relationships between drug-target pairs. Lee et al. [10] developed DeepConv-DTI, a deep learning model employing a 1D-CNN. This model outperforms earlier machine learning models by effectively extracting local residue features from protein sequences using CNN, capturing key local features of proteins more effectively than other protein descriptors. Subsequently, the model employs the extended-connectivity fingerprint (ECFP) [11] algorithm to extract feature information from drugs. However, it does not account for the interaction mechanisms between drugs and proteins, resulting in an inability to capture the interaction patterns between drugs and targets, which impacts the model’s prediction accuracy.

In recent years, researchers have increasingly incorporated various novel attention mechanisms [12] into DTI models to more effectively mine the association information between drugs and proteins. Zhao et al. [13] proposed a specifically designed attention mechanism called HyperAttention, which integrates convolutional neural networks (CNNs) with attention mechanisms to visualize attention scores across spatial and channel dimensions. This approach enables a more comprehensive capture of interaction information between drugs and targets. Huang et al. [14] introduced CoaDTI-pro, a novel interaction feature extraction mechanism. This model consists of stacked cross-attention modules and an encoder-decoder structure, forming a multimodal feature extractor. Although CoaDTI-pro effectively extracts interaction features between drugs and proteins from multimodal data, it exhibits high computational complexity. Shin et al. [15] introduced a pretrained molecular Transformer encoder for drug feature extraction, enabling the model to learn representations of drug molecular structures from extensive molecular data. While improving the model’s ability to comprehend and represent molecular internal data, the computational complexity inherent in the Transformer architecture leads to increased costs during the training and inference phases. Wang et al. [16] employed a heterogenous graph-based algorithmic framework, autonomously extracting useful meta-paths for DTI prediction from heterogenous graphs. This overcomes the dependency on manually defined meta-paths in traditional methods and enhances the adaptability of the algorithm. Although graph neural networks excel in relation detection [17,18], they are less flexible and less efficient when processing large-scale data, making them difficult to scale to large networks. Gong et al. [19] proposed HS-DTI, which utilizes a stacked multi-layer graph neural network to identify and capture specific functional group information in drug molecules, and a CNN module to obtain first and second order sequence information of proteins. The features of proteins and drug molecules are subsequently concatenated to perform predictive tasks. However, this simple cascading operation overlooks cross-modal complementarity and fails to calculate which specific portions of the drug molecule contribute most significantly to the interaction with the target protein.

To address these challenges, we have considered the domain knowledge of substructures, modeling of the molecular overall features, and representation of drug-target interaction relationships. Our proposed model, termed SSCPA-DTI, is a drug-target interaction prediction approach that incorporates substructure subsequences and a cross co-attention mechanism. Through the Substructure Information Mining Module (MIMM), the model extracts substructural features of drugs and proteins, enhancing the granular understanding of critical structural information. Simultaneously, the MIMM algorithm preserves the original features of the drugs and targets, fully utilizing the more global information contained in these original features. Subsequently, a CNN module is used for further feature extraction of the substructural and original features of the drugs/targets. The extracted features pertaining to drugs, proteins, and substructures are subsequently integrated via the cross-co-attention module, followed by the extraction of interaction information. This approach differs from previous models that mechanically concatenate multiple features of drugs/targets, improving the model’s accuracy and interpretability. We compared SSCPA-DTI with other advanced baseline models. Results indicate that SSCPA-DTI performs excellently on three commonly used drug-target datasets.

## Methods

**Fig 1:**
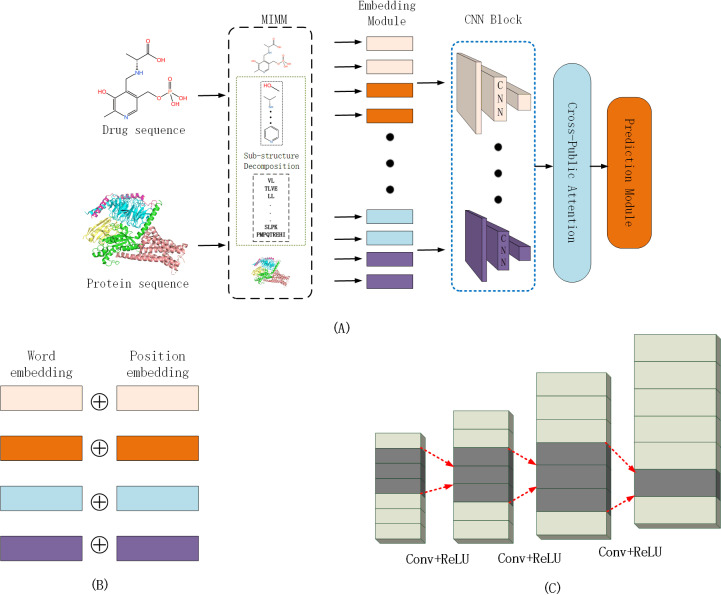
(A) Framework of SSCPA-DTI. (B) Embedding layer: Performs word embedding and positional embedding separately. (C) Convolutional laye.

Fig 1(A) depicts the architectural design of our proposed model, which comprises five distinct components: the Multi-Information Mining Module (MIMM), an embedding layer, a CNN block, a Cross-Co-Attention Module (CPA), and a Fully Connected Network prediction module (FCN).

The core of the model comprises the MIMM and CPA modules. The MIMM module filters substructural features of the drug (or target) while preserving original features, the CNN module further refines features initially extracted by the multi-feature extraction module, and the CPA module is used for feature integration and the extraction of interaction information.

### Multi-feature information mining module

SSCPAT-DTI first orderly decomposes the drug sequences and protein sequences into substructure sequences and preserves their corresponding original sequences. In the domain of natural language processing, the application of subword units [20] has already achieved significant results. We apply these ideas to the mining of substructure information from drug sequences and protein sequences.

Inspired by the BPE (Byte Pair Encoding) algorithm in the field of natural language processing and the PrefixSpan algorithm employed in bioinformatics, we propose a multi-feature information mining module (MIMM) to discover recurrent subsequences in drug and protein databases. MIMM hierarchically decomposes each protein/drug sequence, Decomposing it into subsequences, more diminutive subsequences, and discrete atoms or amino acid symbols, while preserving their corresponding original sequences. We decompose each sequence into a series of orderly discovered frequent subsequences. This process is crucial because these subsequences not only decompose the original sequence but also meet two important conditions: firstly, the union of these subsequences can completely reconstruct each element in the original sequence; secondly, each subsequence is independent of each other without overlapping. The MIMM module is summarized in Algorithm 1.

First, MIMM initializes a set of tokens, denoted as L, for tokenizing protein amino acid/SMILES string characters. Then, with the given set of tokens L, The drugs and proteins each have their own token sets $L_{protein}$ and $L_{drug}$, denoted as, the entire corpus E is tokenized to obtain a tokenized set R, where E can be protein sequences or SMILES sequences from datasets such as Human, C.elegans, KIBA, etc. Next, it iterates through R and identifies the most frequent consecutive tokens (P, Q). Then, MIMM uses the new token (PQ) to update each (P, Q) in the tokenized set R and adds the new token to the token set L. This process is repeated for scanning, identifying, and updating until there are no frequent tokens higher than the threshold d or the size of the vocabulary set L reaches the predefined maximum value ф. Through this process, frequent subsequences are merged into a token, while infrequent subsequences undergo decomposition into a collection of more diminutive tokens, MIMM generates a sequence $\begin{array}{c} M=\left\{A_{1},A_{2},A_{3},\cdot \cdot \cdot A_{k}\right\}\;,B \end{array}$, where each $A_{i}$ is a substructure drug or target protein with a size of k, B is the original sequence of drugs/proteins, and each $A_{i}$ comes from the set L. Through MIMM, input drug and protein sequences can be transformed into sequences of explicit substructures $M_{d}$ and $M_{p}$, as well as original sequences $B_{d}$ and $B_{p}$.

**Table:** 

Algorithm 1 The pipeline of MIMM
**Input:** L denote the collection encompassing all initial amino acids/SMILES tokens; R denote the collection encompassing tokenized proteins/drugs; d symbolizes the predetermined frequency threshold, while θ represents the maximum permissible size for the set V. 1: R ← Using L to tokenize E. 2: **for** t = 1…θ do: 3: (P,Q),freq ← scan R. //(P,Q) is the most frequent consecutive token pair;//freq is its frequency 4: **if** freq < d **then** 5: break //The frequency of (P,Q) is lower than the threshold 6: **end if** 7: R ← find(A,B) ∈ R, replace with(PQ) //update R with the combined token (PQ) 8: R ← L ∪ (PQ) //add (PQ) to the token vocabulary set L 9: **end for**
**Output:** R, the updated tokenized proteins/drugs set; L, the updated token vocabulary set.

**Fig 2:**
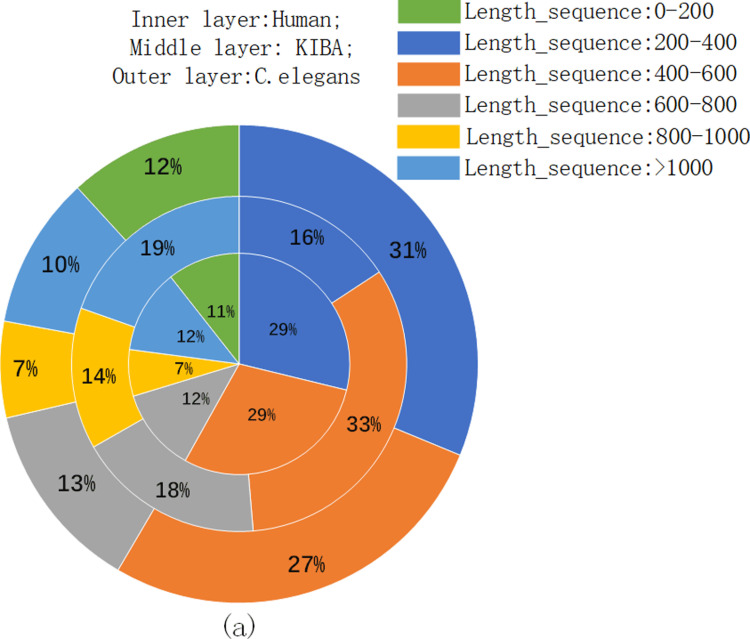
Distribution of protein sequence lengths.

To enable efficient batch training, we investigated the distribution of protein sequence lengths within the dataset, as depicted in Fig 2. We then established a maximum permissible length (MaxL) and implemented either truncation or zero-padding techniques on the respective word embedding matrices.

### Embedding layer

The embedding layer consists of four distinct embedding modules (Fig 1(B)). For every drug-target pair provided as input, we convert the corresponding substructures $M_{p}$, $M_{d}$, and the original sequences $B_{p}$, and $B_{d}$ into four matrices: $C_{p}\in R^{k\times \theta_{p}}$, $C_{d}\in R^{l\times \theta_{d}}$, $Y_{p}\in R^{z\times \omega_{p}}$, $Y_{d}\in R^{g\times \omega_{p}}$.

For the substructural matrices $C_{p}\in R^{k\times \theta_{p}}$ and $C_{d}\in R^{l\times \theta_{d}}$, k/l represents the total size of the protein/drug substructures or the cardinality of the vocabulary set L from the MINN algorithm, while $\theta_{p}$ and $\theta_{d}$ are the maximum lengths of the protein and drug substructure sequences, respectively. Each column $C_{i}^{p}$ and $C_{j}^{d}$ in the matrices are one-hot vectors corresponding to the ith substructure of the protein sequence and the jth substructure of the drug sequence, respectively.

This representation allows the model to effectively capture and distinguish different substructural features, which is crucial for improving prediction accuracy in drug-target interactions.

The content embeddings for proteins and drugs, $E_{{cont}_{i}}^{p}$ and $E_{{cont}_{j}}^{d}$, are generated via learnable dictionary lookup matrices $W_{cont}^{p}\in R^{\vartheta \times k}$ and $W_{cont}^{d}\in R^{\vartheta \times l}$:


$$\begin{array}{c} \begin{array}{c} E_{{cont}_{i}}^{p}=W_{cont}^{p}C_{i}^{p},E_{{cont}_{j}}^{d}=W_{cont}^{d}C_{j}^{d}, \end{array} \end{array}$$ (1)

Where ϑ denotes the dimension of the latent embedding vector corresponding to each substructure. By using learnable matrices, the model can adaptively learn the importance of different substructures, enhancing its ability to model complex interactions between drugs and targets.

Since MIMM uses sequential substructures, we also include position embeddings obtained via query dictionaries $E_{{pos}_{i}}^{p}$, $E_{{pos}_{j}}^{d}$ [21]. These are generated by querying dictionary matrices $W_{pos}^{p}\in R^{\vartheta \times \theta_{p}}$ and $W_{pos}^{d}\in R^{\vartheta \times \theta_{d}}$:


$$\begin{array}{c} E_{{pos}_{i}}^{p}=W_{pos}^{p}\Gamma_{i}^{p},E_{{pos}_{j}}^{d}=W_{pos}^{d}\Gamma_{j}^{d}, \end{array}$$ (2)

Where $\Gamma_{i}^{p}\in R^{\theta_{p}}$/$\Gamma_{j}^{d}\in R^{\theta_{d}}$ is a one-hot vector, with the i/j-th position being 1. This step ensures effective modeling of the positions of elements in the substructure sequence. which is crucial in biological sequences where the relative positioning of features can significantly impact functionality.

The sum of the content and position embedding matrices produces the final embedding matrices $E_{i}^{p}$ and $E_{j}^{d}$:


$$\begin{array}{c} \begin{array}{c} E_{i}^{p}=E_{{cont}_{i}}^{p}+E_{{pos}_{i}}^{p},E_{j}^{d}=E_{{cont}_{j}}^{d}+E_{{pos}_{j}}^{d}, \end{array} \end{array}$$ (3)

This combined representation not only encapsulates the structural information of the substructures but also retains positional context, allowing for enhanced understanding in downstream tasks.

Similarly, for the protein and drug original sequence matrices $Y_{p}\in R^{z\times \omega_{p}}$ and $Y_{d}\in R^{g\times \omega_{p}}$, z/g represents the lengths of the protein/drug sequences, while $\omega_{p}$ and $\omega_{d}$ are the channel dimensions of the embedding vectors for proteins and drugs, respectively.

The content embeddings $G_{{cont}_{i}}^{p}$, $G_{{cont}_{j}}^{d}$ for proteins and drugs are generated by querying learnable dictionary lookup matrices $N_{cont}^{p}\in R^{\delta \times z}$ and $N_{cont}^{d}\in R^{\delta \times g}$, respectively:


$$\begin{array}{c} G_{{cont}_{i}}^{p}=N_{cont}^{p}Y_{i}^{p},G_{{cont}_{j}}^{d}=N_{cont}^{d}Y_{j}^{d}, \end{array}$$ (4)

δ represents the size of the latent embeddings for proteins/drugs. This process allows the model to capture rich representations of the original sequences, facilitating better feature extraction for complex biological interactions. Position embeddings $G_{{pos}_{i}}^{p}$ and $G_{{pos}_{j}}^{d}$ [21] are generated via lookup in the dictionary matrices $N_{pos}^{p}\in R^{\delta \times \omega_{p}}$ and $N_{pos}^{d}\in R^{\delta \times \omega_{d}}$):


$$\begin{array}{c} G_{{pos}_{i}}^{p}=N_{pos}^{p}\Phi_{i}^{p},G_{{pos}_{j}}^{d}=N_{pos}^{d}\Phi_{j}^{d}, \end{array}$$ (5)

$\Phi_{i}^{p}\in R^{\theta_{p}}$ and $\Phi_{j}^{d}\in R^{\theta_{d}}$ are one-hot vectors, where the i-th/j-th position is set to 1. This step reinforces the importance of element positions in the final embeddings, particularly in sequential data where the order of elements is significant.

The sum of the content and position embedding matrices produces the final embedding matrices $G_{i}^{p}$ and $G_{j}^{d}$:


$$\begin{array}{c} G_{i}^{p}=G_{{cont}_{i}}^{p}+G_{{pos}_{i}}^{p},G_{j}^{d}=G_{{cont}_{j}}^{d}+G_{{pos}_{j}}^{d}, \end{array}$$ (6)

This final step integrates both content and positional information, ensuring that the model has a comprehensive representation of each sequence, crucial for effectively modeling the interactions between drugs and targets.

Since proteins, drugs, and their substructure information belong to different feature spaces, our approach employs four independent CNN blocks (Fig 1(C)) focusing on processing drugs, proteins, drug substructure subsequences, and protein substructure subsequences respectively. Each CNN block consists of three consecutive 1D-CNNs, a design efficient in extracting sequence semantic information [22].

For drug and protein sequences, the kernel sizes for each of the three convolutional layers vary, reflecting the distinct structural patterns in proteins and drugs. Specifically, the drug convolutional layers use kernel sizes of 4, 6, and 8, while the protein layers utilize kernel sizes of 4, 8, and 12. This difference in kernel sizes allows the model to capture varying levels of local sequence patterns unique to each molecular type.

The CNN module transforms the input protein embedding matrix $E_{i}^{p}$, drug embedding matrix $E_{j}^{d}$, protein substructure embedding matrix $G_{i}^{p}$, and drug substructure embedding matrix $G_{j}^{d}$ into $P_{CNN}^{ori}\in R^{op\times c},D_{CNN}^{ori}\in R^{o\times c},P_{CNN}^{sub}\in R^{sp\times c},D_{CNN}^{sub}\in R^{s\times c}$ respectively.

### Cross co-attention module

**Fig 3:**
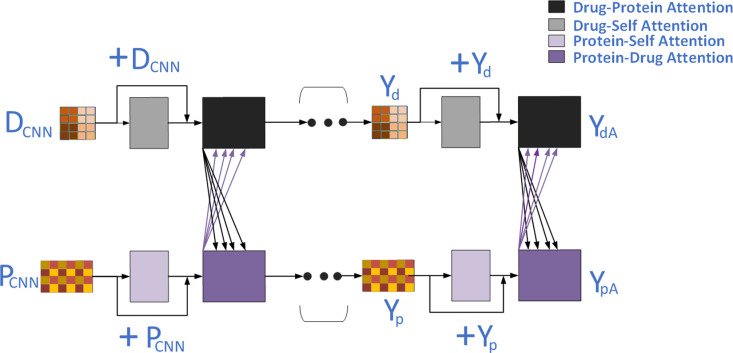
Framework of the cross co-attention mechanism.

Through the preceding modules, we have successfully extracted the original and substructural features of drugs and proteins. Next, we initially integrate the original feature information and substructural features of the drug (or protein), and then extract the representation of interactions between drug targets. Inspired by prior work [23], we constructed a Cross-Co-Attention Module (as shown in Fig 3) in a cascading manner. Its core is composed of stacked modules: DA (Drug Self-Attention), PA (Protein Self-Attention), PDA (Protein-Drug Attention), and DPA (Drug-Protein Attention).

**Fig 4:**
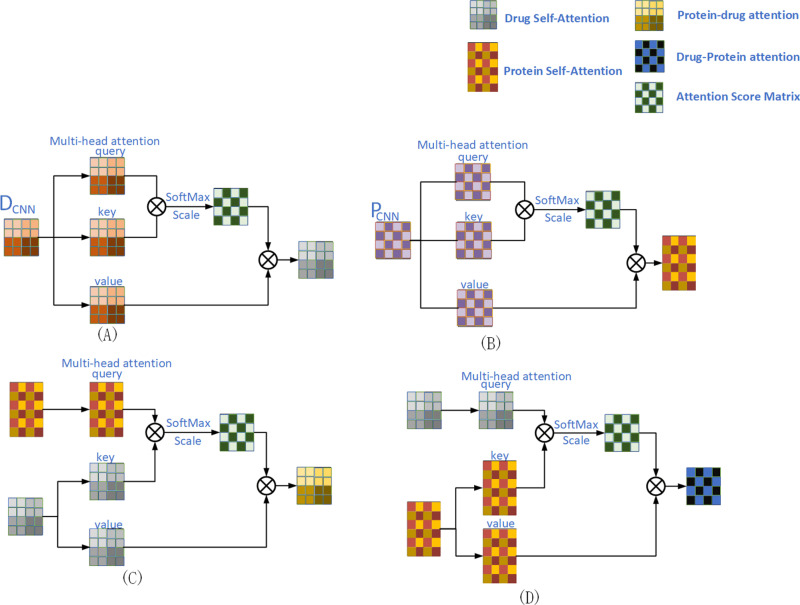
(A) Drug self-attention mechanism. (B) Protein self-attention mechanism. (C) Protein-drug attention mechanism. (D) Drug-protein attention mechanism.

For the input drug or protein features, the DA and PA modules (as shown in Fig 4) are inspired by sequence-to-sequence models, enabling a more intuitive fusion of drug (or protein) original features with substructure features. This approach allows for enhanced flexibility in capturing various interactions, which is crucial in modeling complex biological systems where interactions can vary significantly.

The DA module (Fig 4(A)) first concatenates the input matrices $D_{CNN}^{sub}\in R^{s\times c}$ and $D_{CNN}^{ori}\in R^{o\times c}$ to obtain the drug matrix $D_{CNN}\in R^{d\times c}$. Here, $D_{CNN}^{sub}$ and $D_{CNN}^{ori}$ are the substructure feature matrix and original feature matrix of the drug, respectively. Then, the DA module feeds $D_{CNN}$ into a self-attention mechanism to fuse the original features and substructure features of the drug. The inputs for drug self-attention query, key, and value are computed using the following formulas:


$$\begin{array}{c} \begin{array}{c} Q_{D},K_{D},V_{D}={Linear}_{Q}^{D}\left(D_{CNN}\right),{Linear}_{K}^{D}(D_{CNN}),{Linear}_{V}^{D}(D_{CNN}), \end{array} \end{array}$$ (7)

This linear transformation allows the model to focus on the most relevant features, facilitating the attention mechanism’s ability to highlight critical interactions among the elements of the drug’s representation.

Next, the drug feature fusion matrix is calculated as follows using the softmax function:


$$\begin{array}{c} Attention\left(Q_{D},K_{D},V_{D}\right)=softmax\left(\frac{Q_{D}{K_{D}}^{T}}{\sqrt{d}}\right)V_{D}, \end{array}$$ (8)

Here, $\sqrt{d}$ is used to transform the feature fusion matrix into a standard normal distribution. This normalization step is essential for maintaining numerical stability during training, ensuring that the gradients do not explode or vanish.

Our Cross Co-Attention Module incorporated a multi-head attention mechanism composed of h parallel attention heads. Each of these heads generated a corresponding set of output values, which were subsequently concatenated. This concatenated output then underwent projection, ultimately yielding the drug self-attention matrix $Y_{d}$.


$$\begin{array}{c} \begin{array}{c} MHA\left(Q_{D},K_{D},V_{D}\right)=CONCAT\left({head}_{1},{head}_{2},\cdot \cdot \cdot ,{head}_{h}\right)W^{O},\\ where{head}_{i}=Attention\left(Q_{D}W_{i}^{Qd},K_{D}W_{i}^{Kd},V_{D}W_{i}^{Vd}\right), \end{array} \end{array}$$ (9)

Where $W_{i}^{Qd},W_{i}^{Kd},W_{i}^{Vd}\in R^{d\times d_{h}}$ are projection matrices for the i-th attention head, and $W^{O}\in R^{hd_{h}\times d}$. Here, $d_{h}$ is the output dimension of each attention head. Similarly, the input of the PA module (Fig 4(B)) consists of $P_{CNN}^{sub}\in R^{sp\times c}$ and $P_{CNN}^{ori}\in R^{op\times c}$, resulting in the protein feature matrix $P_{CNN}\in R^{p\times c}$, but the goal is to fuse the original features and substructure features of the protein. The inputs for protein self-attention - query, key, and value - are all calculated from $P_{CNN}\in R^{p\times c}$, and the final protein self-attention matrix is denoted as $Y_{p}$.

The PDA and DPA modules model the spatial and channel dimensions and process the feature matrices through attention mechanisms. They compute cross-attention between drugs and targets to capture their complex interaction relationships, thereby enhancing their feature representation capabilities. This cross-attention mechanism is particularly beneficial in understanding how specific components of a drug influence the behavior of target proteins, allowing for more informed predictions in drug discovery.

The Protein-Drug Attention (PDA) module, illustrated in Fig 4(C), is designed to compute the influence exerted by different components of a drug molecule on the target protein. Specifically, PDA receives two key feature inputs, $Y_{p}$ and $Y_{d}$. Here, $Y_{p}=[y_{p1},\ldots ,y_{pl}]\in R^{l\times d}$ represents the protein feature vector, while $Y_{p}=[y_{p1},\ldots ,y_{pl}]\in R^{l\times d}$ denotes the drug feature vector. The keys and values are derived from $Y_{d}$, whereas the queries are computed from $Y_{p}$. By introducing the multi-head attention mechanism, PDA can learn the complex relationships between $y_{pi}$ and $y_{pi}$ in pairs, and output high-dimensional protein vectors based on the cross-modal similarity of all atomic features between $y_{pi}$ in $Y_{d}$.

Similarly, the role of DPA (Fig 4 (D)) is similar. DPA can effectively measure the effects of different parts of target proteins on drugs. DPA receives $Y_{p}$ and $Y_{d}$ as inputs, generates Keys and values using $Y_{p}$, and calculates queries through $Y_{d}$. This reciprocal attention mechanism facilitates a deeper understanding of how protein features can affect drug interactions, which is vital for predicting drug efficacy and side effects.

The feature representations Y_update, obtained as output from all attention units, are subsequently input into a feedforward layer followed by a dropout layer. Moreover, to bolster the model’s robustness, we incorporated residual connections and normalization techniques.


$$\begin{array}{c} Y=Layernorm\left(Y+Dropout\left(FC\left(Y_{update}\right)\right)\right), \end{array}$$ (10)

This incorporation of dropout layers aids in preventing overfitting, especially in complex models where the risk of memorizing training data is high.

Ultimately, the final attention feature matrices (Y_pA and Y_dA) and the original feature matrices undergo integration via residual connections, yielding the final feature matrix:


$$\begin{array}{c} \begin{array}{c} \left\{ \begin{array}{c} Y_{p}=0.5Y_{pA}+0.5P_{CNN}\\ Y_{d}=0.5Y_{dA}+0.5D_{CNN} \end{array}\;\;\;,\right. \end{array} \end{array}$$ (11)

This final integration allows the model to balance information derived from attention mechanisms and the original feature matrices, providing a comprehensive representation that enhances predictive performance.

### Prediction module

The forecasting component incorporates two maximum pooling layers spanning the entire input, a cascading layer, and one FCN. In this design, global max pooling is applied to the protein feature map $P_{f}$ and the drug feature map $D_{f}$, resulting in 1D feature $D_{pooling}$, both with dimension $R_{dcnn}$. See the equation below:


$$\begin{array}{c} \begin{array}{c} \left\{ \begin{array}{c} D_{pooling}=maxpooling\left(D_{f}\right)\\ P_{pooling}=maxpooling\left(P_{f}\right) \end{array}\;\;,\right. \end{array} \end{array}$$ (12)

The downsampled drug and protein feature vectors are concatenated to form $V_{C}$ (dimension ${2d}_{cnn}\times 1$). See the equation below:


$$\begin{array}{c} \begin{array}{c} V_{C}=Concat\left(D_{pooling},P_{pooling}\right), \end{array} \end{array}$$ (13)

Ultimately, the concatenated feature vector $V_{C}$ is fed into the FCN for DTI prediction. In this module, we use the Leaky Rectified Linear Unit (Leaky ReLU) [24] as the activation function to enhance the model’s ability to express nonlinearity. To effectively address overfitting, we have introduced a Dropout layer following each FCN. The final layer of this output module undertakes the task of representing the likelihood of interaction, outputting a probability value. Considering our task involves binary classification, we have chosen the binary cross-entropy loss function for training the model. The mathematical expression for this loss function is:


$$\begin{array}{c} loss=-\left[ylog\left(\hat{y}\right)+(1-y)\log\left(1-\left(\hat{y}\right)\right)\right], \end{array}$$ (14)

Here, y represents the true label.

Through such a design, we not only preserve the flexibility and non-linearity of the network but also introduce effective mechanisms to prevent overfitting, thus reliably accomplishing the task of binary classification.

## Experiments

During the training process, we followed an 80:20 split to divide the dataset into training and testing sets. Subsequently, the training set was further partitioned into five subsets, with four subsets utilized as training data to train the model, while the remaining subset served as validation data to assess the model’s performance. When the performance of the model on the validation set no longer showed improvement, we proceeded to evaluate its performance on the testing set and retained the corresponding experimental results.

### Datasets

We evaluated our proposed model using three publicly accessible datasets: Human, C.elegans, and KIBA.

The Human and C.elegans datasets were developed by Liu et al. [6]. For these datasets, we employed the construction methodology from CoaDTI [14], ensuring a balanced dataset. The Human dataset includes 3369 positive interactions among 1052 compounds and 852 proteins. The C.elegans dataset comprises 4000 positive interactions involving 1434 compounds and 2504 proteins.

As for the KIBA [25] dataset, it covers information related to kinase inhibitor bioactivity. We applied the dataset construction method from HyperAttentionDTI [13] to create an imbalanced dataset. This KIBA dataset consists of 22,154 positive and 94,196 negative interactions derived from 2068 drugs and 225 proteins.

### Evaluation indicators

To ensure a fair and reasonable comparison with baseline models on the Human and C.elegans datasets, we selected the Area Under the ROC Curve (AUC) as our primary evaluation metric. Additionally, we considered Precision and Recall [14,25,26]. The AUC measures the area under the ROC curve, enclosed by the coordinate axes; a value closer to 1 indicates higher model validity. In the formulas for calculating Precision and Recall, TP are the correctly predicted positive samples, representing the number of drug targets with interactions. FP are positive samples incorrectly predicted. TN are correctly predicted negative samples, representing drug targets without interactions, while FN are negative samples incorrectly predicted.


$$\begin{array}{c} Precision=\frac{TP}{TP+FP}, \end{array}$$ (15)

$$\begin{array}{c} Recall=\frac{TP}{TP+FN}, \end{array}$$ (16)

$$\begin{array}{c} Acc=\frac{TP+TN}{TP+FP+TN+FN}, \end{array}$$ (17)

Additionally, on the KIBA dataset, we employed accuracy (Acc), precision, recall, AUC, and AUPR as metrics to assess the model performance, where AUPR is the area under the precision-recall curve, with a larger area indicating better model performance. The optimal results for each metric will be highlighted in bold to present the model performance on different datasets more clearly.

## Results

### Performance on the C.elegans and Human datasets

**Fig 5:**
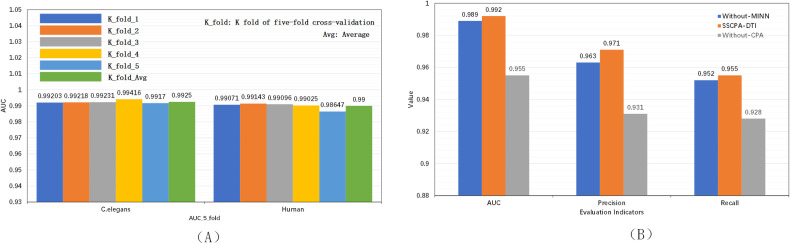
(A) Five-fold cross-validation results on the C. elegans and Human datasets. (B) Ablation experiments of SSCPA-DTI on the C. elegans dataset.

The cross-validation results across five folds for our model, applied to the C.elegans and Human datasets, are depicted in Fig 5(A) to account for potential chance fluctuations. The final outcomes utilize the mean values.

On the C.elegans dataset, as presented in Table 1, we contrast our approach against baseline machine learning models and sophisticated deep learning techniques (including Random Forest (RF), GCN, CPI-GNN [26], MHSADTI [27], TransformerCPI [28], CoaDTI-pro [14], and Wang’s Methodology [29]). Our method exhibits leading performance across the evaluation metrics AUC, Precision, and Recall. Compared to the top-performing baseline models, our approach enhances AUC by 0.4%, Precision by 1.4%, and Recall by 0.2%.

**Table 1:** The results on C.elegans dataset.

Methods	AUC↑	Precision↑	Recall↑
RF	0.902	0.821	0.844
GCN	0.975	0.921	0.927
TransformerCPI	0.988	0.952	0.953
CPI-GNN	0.978	0.938	0.929
MHSADTI	0.983	0.946	0.945
CoaDTI-pro	0.985	0.957	0.948
Wang’s Method	0.987	0.949	0.948
**SSCPA-DTI**	**0.992**	**0.971**	**0.955**

Furthermore, on the Human dataset, as presented in Table 2, our method achieves comparable or superior performance relative to the baseline models, including TransformerCPI, CPI-GNN, GanDTI [30], IIFDTI [31], CoaDTI-pro, and Wang’s Method. Particularly, in terms of AUC and Precision, our method surpasses the optimal performances of all baseline models, showing a 0.6% improvement in AUC and a 1.4% increase in Precision. The slightly lower Recall compared to the baseline models is due to our more cautious approach in predicting samples as positives during model training, thereby reducing false positives (FP).

**Table 2:** The results on Human dataset.

Methods	AUC↑	Precision↑	Recall↑
TransformerCPI	0.973	0.916	0.925
CPI-GNN	0.970	0.918	0.923
GanDT	0.983	0.933	**0.960**
IIFDTI	0.984	0.946	0.947
CoaDTI-pro	0.982	0.952	0.950
Wang’s Method	0.982	0.931	0.936
**SSCPA-DTI**	**0.990**	**0.955**	0.946

### Performance on the KIBA datasets

Ultimately, we applied and evaluated our proposed methodology on the KIBA dataset, conducting comparative trials against baseline models.

Table 3 outlines the results in detail. The KIBA dataset exhibits a pronounced category imbalance, presenting substantial hurdles that commonly impede the performance of deep neural networks. Nonetheless, our approach surpassed the peak performance of the baseline models across metrics like AUC, AUPR, ACC, and Precision.

**Table 3:** The results on KIBA dataset.

Methods	AUC↑	AUPR↑	ACC↑	Precision↑	Recall↑
DeepConv-DTI	0.8980	0.7030	0.8780	0.7080	0.6360
TransformerCPI	0.9070	0.7648	0.8850	0.7200	0.6598
MolTransDTI	0.9232	0.7949	0.8891	0.7042	**0.7353**
HyperAttentionDTI	0.9161	0.7721	0.8775	0.6730	0.7149
**SSCPA-DTI**	**0.9237**	**0.8010**	**0.8969**	**0.7493**	0.7004

### Ablation experiments

**Fig 6:**
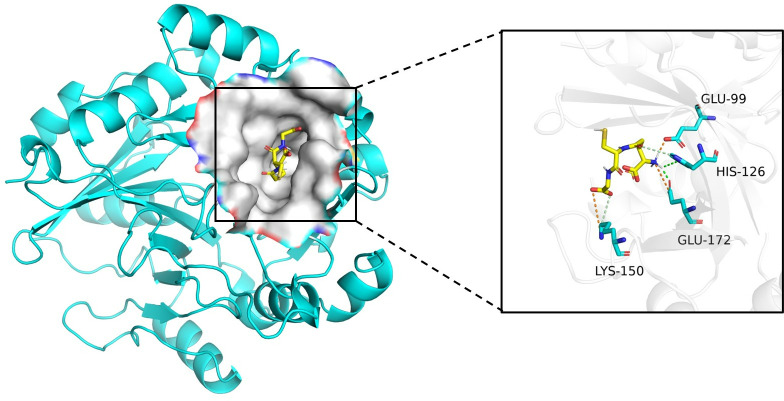
We plotted the hydrogen bond interactions and electrostatic interactions in the interaction mode between glutathione (DB00143) and gamma-glutamyl hydrolase (Q04760). Green dashed lines represent hydrogen bond interactions, and red dashed lines represent electrostatic interactions.

To further validate the efficacy of our proposed methodology, we performed ablation experiments on the C.elegans dataset. These experiments targeted both the multi-feature information extraction module and the cross co-attention module. Initially, we removed the multi-feature information mining module from SSCPA-DTI, created a variant model called Without-MINN, and compared it with SSCPA-DTI to verify the effectiveness of the multi-feature information mining module. As shown in Fig 6, the multi-feature information mining module improved accuracy by 0.04 (4.29%), AUC by 0.037 (3.87%), and Recall by 0.027 (2.90%). This indicates the effectiveness of the multi-feature information mining module in significantly enhancing model performance. Secondly, we removed the cross co-attention module from SSCPA-DTI, inputted the extracted drug and target features into the prediction module to form another model named Without-CPA, and compared it with SSCPA-DTI to validate the importance of the cross co-attention module. As illustrated in Fig 6, the cross co-attention module increased accuracy by 0.003 (0.31%), AUC by 0.003 (0.3%), and Precision by 0.008 (0.83%). These results further emphasize the crucial role of the cross co-attention module in enhancing model performance.

### Case study

We randomly selected a drug and its interacting target protein from the DrugBank dataset, and then used a pre-trained model to predict their interactions. Glutathione (DB00143) is the drug we randomly selected. Glutathione plays an important role in detoxification processes, as it can bind to some toxic substances or metabolites to help cells eliminate them. It can interact with targets such as glutathione S-transferase (Q04760). Table 4 shows the interaction results between glutathione and Q04760, as well as several other targets. For glutathione (DB00143), we had one incorrect prediction out of 10 positive samples and only two incorrect predictions out of 10 negative samples, resulting in an accuracy of 85%.

**Table 4:** Prediction result of drug Glutathione.

Drug	Protein	Prediction	Real
**DB00143**	**Q04760**	**True**	**True**
	**Q16873**	**True**	**True**
	**P36969**	**True**	**True**
	**O60760**	**False**	**True**
	**P28161**	**True**	**True**
	**Q16775**	**True**	**True**
	**P09210**	**True**	**True**
	**O14880**	**True**	**True**
	**O95881**	**True**	**True**
	**P08263**	**True**	**True**
	**Q9NQX3**	**False**	**False**
	**Q9UDR5**	**False**	**False**
	**P00813**	**False**	**False**
	**P45059**	**True**	**False**
	**P19113**	**False**	**False**
	**Q9UI32**	**False**	**False**
	**P00488**	**False**	**False**
	**P35228**	**False**	**False**
	**P37059**	**False**	**False**
	**P06737**	**True**	**False**
**Accuracy**			**85%**

As shown in Fig 6, we retrieved glutathione S-transferase from the PDB database. We conducted molecular docking of DB00143 and Q04760 using software such as PYMOL, AutoDockTools, and AutoDock Vina. The docking results were visualized using Discover Studio and PyMOL. The docking free energy score between Q04760 and DB00143 is -4.9 kcal/mol. The small molecule forms hydrogen bonds with HIS126 and GLU172, and electrostatic interactions with LYS150, GLU99, and GLU172.

The aforementioned experimental findings highlight that our proposed approach exhibits strong predictive capabilities and generalization competence when forecasting drug-target interactions.

## Conclusion

We propose a DTI prediction model based on substructure subsequences and a cross-coattention mechanism, integrating multi-feature information to predict DTIs. It extracts features from drug and protein sequences, including substructural features of drug-target interactions and original features; substructural information provides detailed insights into the local molecular structures, while original features encompass more global molecular characteristics. The cross co-attention module first merges the extracted original and substructural feature information, then captures the interactive data between the proteins and drugs. By integrating both original and substructural feature information, enhancing the model’s understanding of the overall molecular structure and enabling it to better differentiate molecules with similar global structures but distinct substructures. Across all experimental configurations, the outcomes showcase that our model exhibits remarkable proficiency regarding metrics such as AUC, Precision, and other evaluation criteria.

## Supporting information

Fig 1 celegans_train.csv.
(CSV)

Fig 2 celegans_test.csv.
(CSV)
